# Characteristics and risk factors for advanced lung cancer with pulmonary embolism: A cross‐sectional, case–control study

**DOI:** 10.1111/crj.13692

**Published:** 2023-09-07

**Authors:** Yongkang Huang, Shiyuan Gao, Ting Li, Beilei Zhang, Juan Du, Yajuan Qian, Yufei Xing, Tong Zhou, Minhua Shi, Jian‐an Huang, Yixin Lian

**Affiliations:** ^1^ Department of Respiratory and Critical Care Medicine The Second Affiliated Hospital of Soochow University Suzhou Jiangsu China; ^2^ Department of Respiratory and Critical Care Medicine The First Affiliated Hospital of Soochow University Suzhou Jiangsu China

**Keywords:** characteristic, lung cancer, prevalence, risk factors, survival

## Abstract

**Objectives:**

Pulmonary embolism (PE) is a life‐threatening complication that can occur in patients with lung cancer. In this study, we aimed to identify risk factors and examine the clinical characteristics of advanced lung cancer patients with PE.

**Methods:**

We conducted a retrospective review of patients admitted to our two hospitals between January 2020 and June 2022. The case group consisted of patients with lung cancer and PE, and a closely matched control group was included to identify risk factors. Statistical analysis was conducted using R language.

**Results:**

A total of 4957 patients were reviewed, and 162 patients (comprising 54 cases and 108 controls) were included in this study. The prevalence of lung cancer with PE in the study population was 1.08%. The majority of patients were male, and the most common histological subtype was adenocarcinoma (67%), followed by squamous cell carcinoma, small cell carcinoma, and poorly differentiated non‐small cell lung cancer. The majority of patients had a high performance status (PS) score, with 50% experiencing respiratory failure (mainly hypoxia) and 33% with deep vein thrombosis (DVT). Forty‐eight percent of patients were diagnosed with concurrent PE. Further analysis showed that PE was an independent predictor of poor survival, and a PS score of >1 was an independent risk factor for PE in patients with lung cancer.

**Conclusion:**

Our study provides valuable insights into the epidemiology and prognosis of PE in lung cancer patients and suggests that a poor ECOG PS, which has not been previously reported, is an independent risk factor for PE.

## Graphical abstract

This study examined the clinical characteristics and potential risk factors of pulmonary embolism (PE) in advanced lung cancer patients. Notably, dyspnea was the most common complaint in lung cancer patients with PE, while a poor performance status (PS) score was found to be an independent risk factor for PE in lung cancer patients.

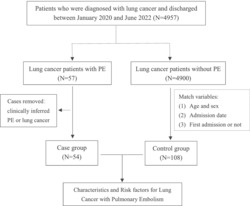

## 1 INTRODUCTION

Pulmonary embolism (PE) is a life‐threatening syndrome caused by an embolism in pulmonary arteries and/or their branches, usually resulting from detachment of deep venous thrombosis (DVT). According to epidemiological studies, the current annual incidence rates of PE in Europe have been reported to reach as high as 115 per 100 000 population.^1^ In some cases, it can lead to sudden death.^2^ Patients with cancer are at an increased risk for both the development of PE and the development of more severe forms of the disease. The overall risk of venous thromboembolism (VTE), which consists of DVT and PE, in patients with malignant tumors was reported to be nearly seven times higher than in those without cancer.^3^ As one of the most common cancers worldwide, lung cancer was reported to be the most frequent cancer susceptible to PE,^4^, ^5^, ^6^, ^7^ accounting for 23.5%^4^ of PE patients with malignancy. Moreover, lung cancer patients with PE were at higher risk of in‐hospital death compared to patients with PE and other malignancies combined.^4^

Published studies have shown that most lung cancer patients with PE present in an advanced stage, and dyspnea is one of the most frequent complaints in these patients. High D‐dimer,^8^, ^9^ chemotherapy,^8^ presence of DVT,^8^ a TNM stage of III–IV,^8^, ^9^ adenocarcinoma,^8^, ^9^ ALK mutation,^10^, ^11^, ^12^ and positive PD‐L1 expression^12^ were associated with an increased risk of PE in lung cancer patients. However, these studies featured small sample sizes or were cross‐sectional studies without a control group or were based on pan‐cancer studies, and the characteristics and risk factors of lung cancer with PE were not sufficiently reported. In this study, we described the clinical presentation, characteristics of clinical tests, treatment, and prognosis of lung cancer patients with PE from two tertiary hospitals and compared them with a matched control group to further identify risk factors.

## 2 MATERIALS AND METHODS

**FIGURE 1:**
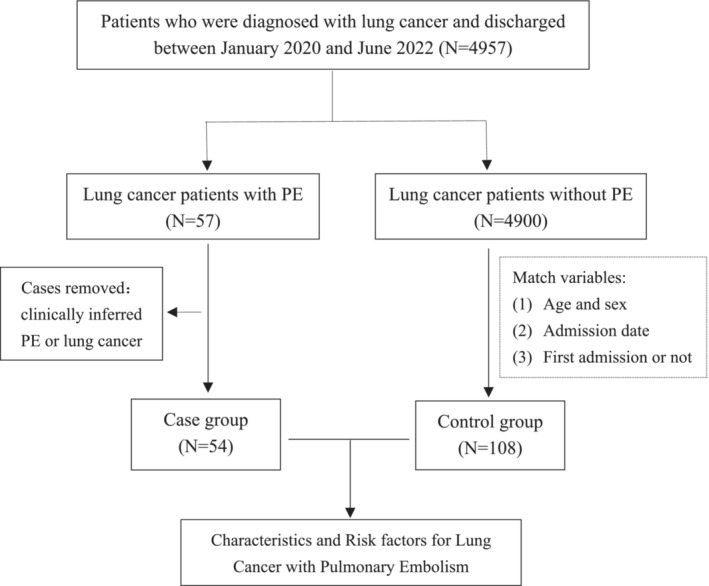
Study flow.

This was a two‐center, retrospective study. The study was reported as per the STROBE statement.^13^ The study flow is shown in Figure 1.

### 2.1 Study population

Patients who were admitted into our hospitals from January 2020 to June 2022 and diagnosed with lung cancer were retrospectively reviewed. To be included in the case group, patients had to meet the following criteria: (1) Lung cancer should be diagnosed pathologically, and (2) PE should be diagnosed based on contrast‐enhanced chest CT or CT pulmonary angiogram (CTPA) by at least two radiologists. Patients with clinically inferred PE or lung cancer were excluded. If multiple admissions met the criteria, the first hospitalization of the patient was preferred. For each case identified, two control patients without PE matched according to age, sex, whether it was the patient's first admission for lung cancer or not, and admission date (within 1 year) were enrolled consecutively to identify risk factors.

### 2.2 Clinical data

The following data were collected, which included (1) demographic and baseline data such as age, gender, body mass index (BMI), pathological type of lung cancer, smoking history, prophylactic anticoagulation and symptoms; (2) blood test results including arterial blood gas analysis, biochemical testing, blood routine, D‐Dimer, serum carcinoma embryonic antigen (CEA), serum cytokeratin 19 (also known as CYFRA 21‐1), and serum neuron‐specific enolase (NSE); (3) equipment inspections such as ultrasound scanning, CT scanning, and electrocardiogram; (4) treatment‐related information including anticoagulation therapy, whether a central venous catheterization was conducted, whether chemotherapy, antiangiogenic therapy, or immunotherapy was administered; and (5) prognosis data focusing on whether the PE was resolved detected by contrast‐enhanced chest CT or CTPA and the overall survival (OS) of the patients. In the abovementioned data, we collected the pretreatment data at the time of admission for those who had PE at the first admission. For those who developed PE during the follow‐up period for lung cancer, we collected the data before or at the initial PE diagnosis. Survival data for the patients in this study were obtained through telephone follow‐up. The follow‐up process began 2 months after the patients' discharge and continued every 2 months thereafter. The last follow‐up was conducted on December 31, 2022.

### 2.3 Statistical analysis

Statistical analyses were carried out by R (version 4.1.1) or SPSS (version 22.0). Frequency and proportion were reported for dichotomous data, and the chi‐square test was used for hypothesis testing. Continuous variables were reported as mean and SD or median and interquartiles, and were tested using *t*‐tests or Wilcoxon signed rank tests where applicable. Survival characteristics were visualized using the Kaplan–Meier curve, and the relationships between variables and survival were tested and adjusted by Cox regression analysis. Considering the limited case sample, only those variables that were well‐acknowledged factors or got a *p*‐value of no more than 0.05 on univariate conditional logistic regression analysis were subjected to multivariate conditional logistic regression models when exploring the risk factors for lung cancer with PE. A *p*‐value of less than 0.05 was used to indicate statistical significance.

## 3 RESULTS

### 3.1 Characteristics of lung cancer with PE

A total of 4957 patients diagnosed with lung cancer were reviewed; 57 patients with PE were retained for further assessment, and 54 patients (1.08%) were finally included in the case group. Subsequently, 108 lung cancer patients without PE were enrolled correspondingly in the control group as mentioned before. The study flow is shown in Figure 1.

The cases had a mean age of 67 ± 10 years, and a mean BMI of 22.80 ± 2.54 kg/m^2^, and all cases were in an advanced TNM stage (III–IV). Male patients were in the majority, making up about 67% (*n* = 36/54) of the population, and smokers or ex‐smokers accounting for about 46% (*n* = 24/54). Adenocarcinoma was the predominant pathological type, responsible for 67% of cases (*n* = 36/54), followed by squamous carcinoma (22%, *n* = 12/54), small cell carcinoma (7.4%, *n* = 4/54) and poorly differentiated cancer (3.7%, *n* = 2/54). The patients with an ECOG PS score greater than or equal to 2 points were 28 (52%), and 21 cases (39%) of patients were with chronic airway disease including chronic obstructive pulmonary disease (COPD), asthma, or bronchiectasis. The characteristics of cases and controls are summarized in Table 1.

**TABLE 1:** The demographics and characteristics of patients.

Variable	Non‐PE, *N* = 108	PE, *N* = 54
Sex		
Female	37/108 (34%)	18/54 (33%)
Male	71/108 (66%)	36/54 (67%)
Age (years)	67 (10)	67 (10)
Histological type		
LUAD	67/108 (62%)	36/54 (67%)
LUSC	19/108 (18%)	12/54 (22%)
SCLC	20/108 (19%)	4/54 (7.4%)
Poorly differentiated NSCLC	2/108 (1.9%)	2/54 (3.7%)
BMI	22.81 (3.25)	22.80 (2.54)
Driver gene mutation		
Positive	39/108 (36%)	17/54 (31%)
Negative	37/108 (34%)	18/54 (33%)
Not Available	13/108 (12%)	15/54 (28%)
Not applicable	19/108 (18%)	4/54 (7.4%)
Smoking history	61/108 (56%)	25/54 (46%)
Chronic lung disease	34/108 (31%)	21/54 (39%)
Hypertension	46/108 (43%)	25/54 (46%)
Diabetes	18/108 (17%)	9/54 (17%)
Prophylactic anticoagulation	2/108 (2%)	0/54 (0%)
HB (g/L)	126 (114, 136)	123 (102, 138)
PLT (× 10^9^/L)	230 (95)	208 (85)
WBC (× 10^9^/L)	6.40 (4.98, 8.00)	7.64 (5.85, 9.40)
ALT (U/L)	19 (13, 27)	23 (15, 39)
AST (U/L)	23 (18, 30)	26 (19, 40)
Cr (μmol/L)	63 (54, 76)	60 (53, 78)
D‐Dimer	0.72 (0.39, 1.33)	3.98 (1.79, 13.81)
CEA	57/108 (53%)	29/54 (54%)
CYFRA 21‐1	56/108 (52%)	39/54 (72%)
NSE (positive)	34/108 (31%)	25/54 (46%)
PS score		
1	94/108 (87%)	26/54 (48%)
2	13/108 (12%)	14/54 (26%)
3	1/108 (0.9%)	14/54 (26%)
Central venous catheterization	44/108 (41%)	20/54 (37%)
Chemotherapy	48/108 (44%)	20/54 (37%)
Antiangiogenesis therapy	25/108 (23%)	10/54 (19%)
Immunotherapy	27/108 (25%)	7/54 (13%)
Overall survival	17 (9, 28)	10 (3, 21)

*Note*: Variables are expressed as *n*/*N* (%), or mean (SD); median (lower quartile, upper quartile); median (IQR).

**FIGURE 2:**
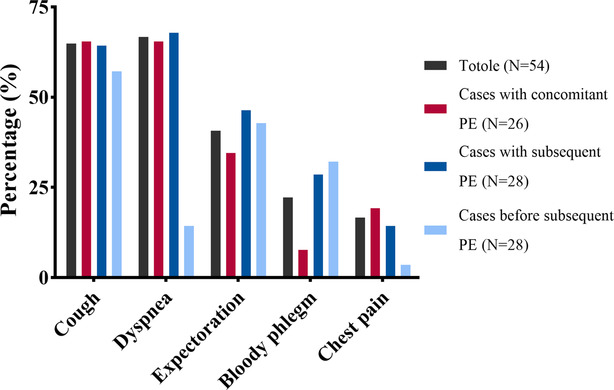
Symptoms of lung cancer patients with pulmonary embolism. The cases were divided into two groups. The cases with concomitant pulmonary embolism (PE) subgroup included those who were diagnosed with PE at the same time as lung cancer, while the others were grouped into cases with subsequent PE. Symptoms were accordingly collected and summarized.

At the time of lung cancer diagnosis, 26 patients (48%) were diagnosed with concurrent PE. Of these cases, 25 were symptomatic with cough (*n* = 17, 65.4%) and dyspnea (*n* = 17, 65.4%) being the most prominent complaints. Other symptoms included expectoration (*n* = 9, 34.6%), chest pain (*n* = 5, 19.2%), and bloody phlegm (*n* = 2, 7.6%). Only one patient was asymptomatic and PE was incidentally diagnosed on routine enhanced CT scanning. However, 28 patients developed PE during follow‐up, with the median time for diagnosis being 14 months. Most of these cases (*n* = 19, 67.8%) were characterized by dyspnea as the primary symptom, while four cases were characterized by confusion, and three cases were asymptomatic. The symptoms of the patients are summarized and visualized in Figure 2.

### 3.2 Arterial blood gas analysis

There were 34 cases of patients who completed arterial blood gas analysis, with half of them (*n* = 17/34) developing respiratory failure. Among those complicated with respiratory failure, 16 presented with hypoxic respiratory failure, while one case had hypoxemia and hypercapnia. Nine patients had elevated lactic acid levels. In the subgroup with respiratory failure, eight were classified as having intermediate‐risk PE.

### 3.3 Gene mutation patterns

For patients with non‐small cell lung cancer (NSCLC), molecular testing is usually recommended to identify those who harbor positive driver gene mutations and may benefit from targeted therapy.^14^, ^15^ In our study, 35 of 50 cases of NSCLC patients underwent next‐generation sequencing (NGS) analysis, and EGFR, ALK, and ROS1 mutation patterns were collected. Of these, 13 and 3 cases harbored a common mutation in the EGFR and ALK genes, respectively, while no ROS1 mutations were found.

### 3.4 Blood tests

Fifty‐one patients in the case group completed tumor marker tests, revealing increases in CEA, CYFRA 21‐1, and NSE in 28, 37, and 19 patients, respectively. Additionally, 52 patients had NT‐proBNP and troponin T (TnT) tests, which revealed elevated NT‐proBNP in 15 cases and elevated TnT in 13.

### 3.5 Auxiliary inspections

Of the 54 cases, 38 underwent Doppler ultrasonography of the lower extremities, revealing 13 cases (34.2%) with deep vein thrombosis (DVT) and nine (23.6%) with superficial vein thrombus, and no thrombus detected in the remaining cases (42.1%).

A cardiac echocardiogram was performed in 30 patients, with a median ejection fraction of 65% (range: 39.11%–79%). Pulmonary hypertension was observed in 11 cases, and six cases displayed acute dilated right atrium and/or ventricle. Electrocardiograms (ECG) were available for 20 patients, with most showing no abnormality. T‐wave changes, right bundle branch block, S_I_Q_III_T_III_, and sinus tachycardia were noted in eight, four, two, and two patients, respectively. Premature contraction, atrial fibrillation, and sinus arrhythmia each occurred in one patient.

### 3.6 Treatments and outcomes

Stratified by classification of PE severity,^1^ the majority of patients (31/54) were in the low‐risk group, and 23 of 54 in the intermediate‐risk group. None of the patients received thrombolytic therapy; instead, 51 of 54 received anticoagulant therapy following confirmation of PE. Of these, 49 out of 51 were treated with low molecular weight heparin when hospitalized and received oral anticoagulant therapy when discharged, while two patients were administered rivaroxaban throughout the treatment. Three patients were unable to receive anticoagulation due to contraindications (one complicated with gastrointestinal bleeding, two with high blood risk and low platelet count).

Twelve patients died during their hospitalization, while 42 were discharged after their symptoms improved and were prescribed oral anticoagulants as sequential therapy at discharge: 38 were given direct oral anticoagulants and four warfarin. Of the 42 patients, chest CT or CTPA data were available for review in 16 during follow‐up. Anticoagulation therapy was found to be effective in 10 cases, with embolism removed from the lung vessels in three cases within 1 month, four within 3 months, and three within 6 months. Thrombus shrunk in four cases, but two experienced repeated aggravation and improvement.

### 3.7 The impact of PE on the survival of lung cancer patients

**FIGURE 3:**
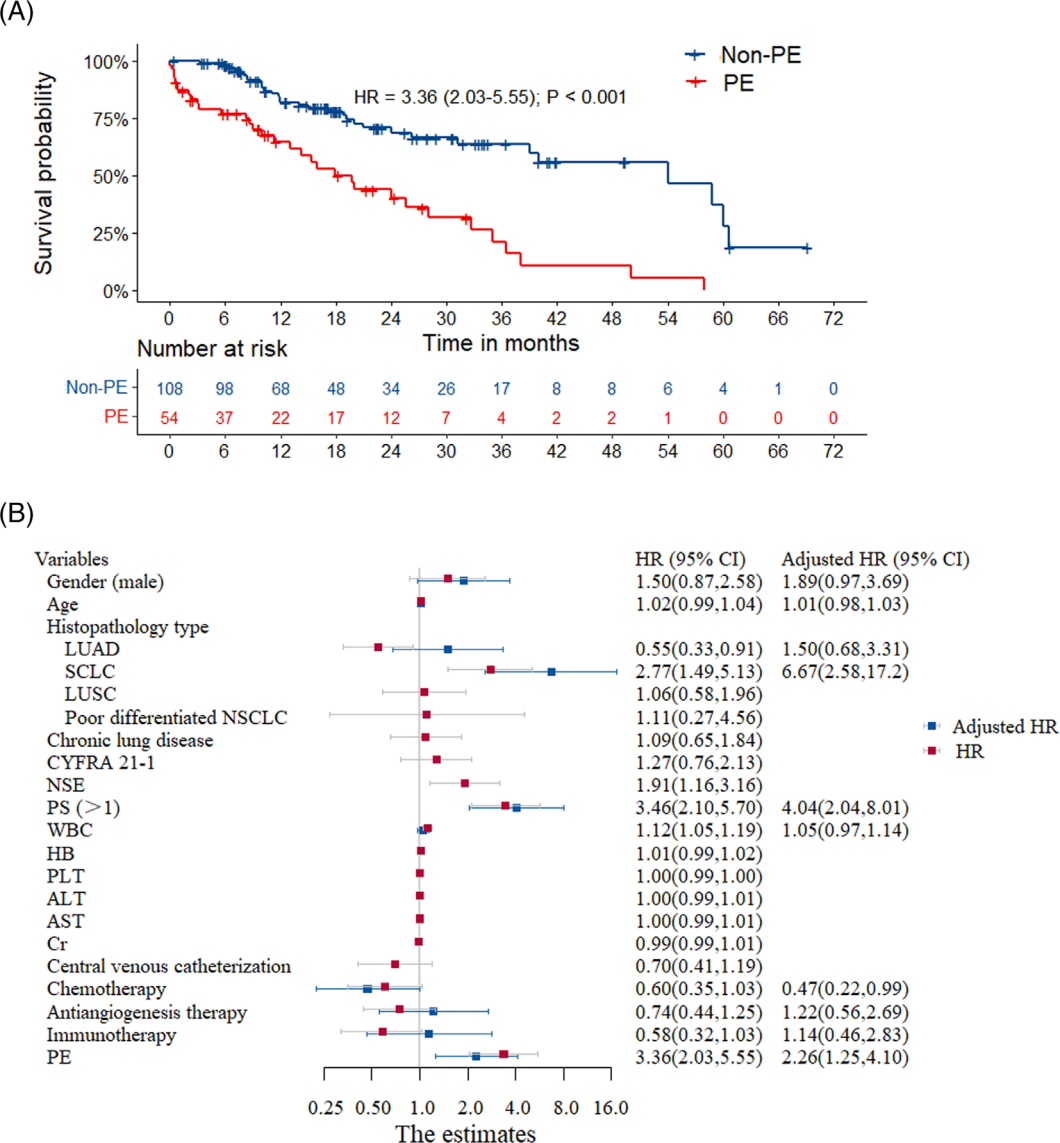
Survival curve and risk factors to survival of lung cancer patients. (A) The Kaplan–Meier plot that compares the overall survival (OS) of patients with lung cancer who were classified into two groups, PE and non‐PE, based on their association with pulmonary embolism. (B) The forest plots that depict the results of univariate and multifactor cox regression analyses of the factors that potentially impact the OS of the patients.

In order to explore the influence of PE on the prognosis of lung cancer patients, 108 patients without PE were enrolled in the control group as previously described. The Kaplan–Meier curve (shown in Figure 3A) showed that patients with PE had significantly shorter median survival than those without PE (19.7 months vs. 54.0 months, log‐rank test *p* < 0.001). Moreover, univariate Cox regression analysis revealed that PE was associated with survival (unadjusted HR = 3.36, 95% CI 2.03–5.55, *p* < 0.001), as well as pathological type of lung cancer, NSE, white blood cell (WBC) count, and ECOG PS score (all *p* < 0.05). Subsequent multivariate Cox regression analysis further confirmed that PE was an independent prognostic factor (adjusted HR = 2.26, 95% CI 1.25–4.10, *p* < 0.001), after adjusting for age, sex, pathological type of lung cancer, NSE, WBC count, chemotherapy, antiangiogenesis therapy, immunotherapy, and ECOG PS score, which is consistent with the previous analysis. The findings of the analyses are visualized by forest plot shown in Figure 3B.

### 3.8 Risk factors of lung cancer with PE

We further investigated the association between easily obtainable clinical variables and the onset of PE. These variables were subjected to univariate conditional logistic regression analysis, including smoking history, BMI, histopathology types, complications (chronic lung disease, hypertension, diabetes), tumor markers (CEA, CYFRA 21‐1, NSE), treatment‐related characteristics (central venous catheterization, chemotherapy, anti‐vascular, immunotherapy), and ECOG PS score. The results suggested that smoking history (OR = 0.324, 95% CI 0.106–0.986, *p* = 0.047) and immunotherapy (OR = 0.305, 95% CI 0.097–0.964, *p* = 0.043) were associated with lower odds of PE, while CYFRA 21‐1 (OR = 2.427, 95% CI 1.189–4.956, *p* = 0.014), WBC (OR = 1.223, 95% CI 1.081–1.383, *p* = 0.001), AST (OR = 1.017, 95% CI 1.001–1.035, *p* = 0.043), and PS score of more than 1 (PS > 1) (OR = 10.849, 95% CI 3.752–31.37, *p* < 0.001) were associated with higher odds of PE. However, the multivariate conditional logistic analysis indicated that only the PS score was a significant independent risk factor for lung cancer with PE (adjusted OR = 11.40, 95% CI 3.28–39.70, *p* < 0.001). The findings of the analyses are summarized in Table 2.

**TABLE 2:** Correlation between potential predictors and lung cancer with PE by univariate and multivariate conditional logistic regression analysis.

Variables	Univariate logistic regression			Multivariate logistic regression		
	HR	95% CI	*p*‐value	Adjusted HR	95% CI	*p*‐value
Sex (male)	1.69	(0.14, 21.13)	0.685			
Age	0.93	(0.75, 1.17)	0.549			
LUAD	1.26	(0.60, 2.63)	0.539			
LUSC	1.40	(0.58, 3.35)	0.453			
SCLC	0.35	(0.11, 1.08)	0.067			
Poor differentiated NSCLC	2.00	(0.28, 14.20)	0.488			
BMI	1.00	(0.90, 1.11)	0.977			
Smoking history	0.32	(0.11, 0.99)	0.047	0.31	(0.07, 1.50)	0.147
Chronic lung disease	1.65	(0.70, 3.88)	0.252			
Hypertension	1.18	(0.59, 2.33)	0.642			
CEA	1.04	(0.55, 1.94)	0.915			
CYFRA 21‐1	2.43	(1.19, 4.96)	0.015	1.41	(0.55, 3.60)	0.477
NSE	1.82	(0.94, 3.54)	0.077			
Central venous catheterization	0.61	(0.18, 2.03)	0.418	3.33	(0.51, 21.80)	0.209
Chemotherapy	0.37	(0.11, 1.27)	0.114			
Antiangiogenesis	0.73	(0.30, 1.74)	0.472	0.93	(0.27, 3.17)	0.905
Immunotherapy	0.31	(0.10, 0.97)	0.043	0.24	(0.06, 1.03)	0.056
WBC	1.22	(1.08, 1.38)	0.001	1.15	(0.98, 1.35)	0.087
HB	1.00	(0.98, 1.01)	0.497			
PLT	1.00	(0.99, 1.00)	0.174			
AST	1.02	(1.00, 1.04)	0.043	1.02	(0.99, 1.04)	0.174
ALT	1.01	(0.99, 1.02)	0.077			
Cr	1.00	(0.98, 1.01)	0.880			
PS(> = 1)	10.85	(3.75, 31.37)	0.001	11.40	(3.28, 39.7)	<0.001

Abbreviations: CI, confidence interval; HR, hazard ratio.

## 4 DISCUSSION

DVT was the primary cause of PE, which necessitated Virchow's Triad comprising stagnation of blood flow, endothelial injury, and hypercoagulability.^16^ Malignant tumors, such as lung cancer, have been observed to elicit cytokine secretion from immune cells^17^ while simultaneously releasing fibrinolytic inhibitors, ultimately leading to a state of hypercoagulability.^18^ Moreover, tumor cells can directly invade blood vessels, damage the endothelium, and eventually result in the formation of thrombi. In this study, we examined the occurrence of lung cancer in combination with PE in our region. We conducted a comprehensive analysis of the clinical characteristics of these patients and found it is not uncommon to see patients with PE at the first diagnosis of lung cancer. In addition, a poor ECOG PS, which has not been previously reported, is an independent risk factor for PE.

Enhanced CT is routinely used in our two clinical centers to aid in further biopsy specimens and TNM staging for patients suspected of having lung cancer. As a result, we are confident in our ability to accurately detect PE in lung cancer patients. In this retrospective study, we found that the prevalence of PE in lung cancer patients was 1.08%. Although the rate is similar, it is slightly higher than that reported by Tsubata et al (0.62%)^19^ and Awano et al (0.6%).^20^ This difference could be attributed to the racial composition of our study population and the higher proportion of patients with advanced TNM stages.

Our study found that lung cancer patients with PE had a significantly reduced OS and that PE was an independent risk factor for poor prognosis in lung cancer. Therefore, identifying lung cancer patients who are at high risk for PE is crucial for determining appropriate treatment. Unfortunately, the conventional D‐dimer cutoff value in our population had good sensitivity (98.1%) but poor specificity (36.1%). The specificity was improved at 48.1% using age‐adjusted D dimer,^16^ but this is still unsatisfactory. Furthermore, half of the lung cancer patients with PE did not develop respiratory failure, and hypoxia was a more prominent feature in other patients. Our study also revealed that lower extremity vascular ultrasound was not sufficiently sensitive to detect PE, with nearly two‐thirds of patients showing no deep vein thrombosis. While ECG and echocardiography had limited diagnostic value, PE should still be considered if relevant results are present. Dyspnea was a prominent complaint among our patients, while chest pain and hemoptysis were less frequent. When lung cancer patients present with dyspnea that cannot be attributed to their cancer, especially if accompanied by hypoxia or respiratory failure, PE should be suspected.

Previous literature showed that adenocarcinoma was the most common subtype of lung cancer with PE^9^, ^21^ and that it was also a risk factor for PE.^8^ In this study, adenocarcinoma was indeed the most frequent pathological type associated with PE, accounting for approximately 67% of cases. However, we did not observe a significant difference in the proportion of adenocarcinoma between the case group and control group, suggesting that it may not be a risk factor. This may be explained by the fact that adenocarcinoma is the most common histopathological subtype of lung cancer^22^ and has a high prevalence among patients with lung cancer. Similar results were also found in the study of Liu et al^9^ (28/53 vs. 23/53, *p* = 0.3311) and Xiong et al^23^(46.7% vs. 43.6%, *p* = 0.33) where no significant difference was found in the incidence of PE between the adenocarcinoma group and the control. Interestingly, small cell lung cancer seems to be less frequently associated with PE, but the intergroup difference did not reach statistical significance (OR = 0.35, 95% CI 0.11–1.08, *p* = 0.067).

Several studies have shown that positive PD‐L1 is correlated with the occurrence of PE in NSCLC patients,^12^, ^24^, ^25^ suggesting that immunotherapy targeting PD‐1/PD‐L1 axis might have a protective effect against PE. A retrospective cohort study also showed a reduced VTE incidence for patients receiving immune checkpoint inhibitors (ICI) based regimens compared with those receiving chemotherapy‐based and ICI + chemotherapy regimens.^26^ In our study, immunotherapy served as a protective factor against lung cancer with PE in univariate analysis; however, it did not reach significance in multivariate logistic analysis. Moreover, a recent meta‐analysis found no significant difference in VTE risk between ICI and non‐ICI regimens.^27^ The observation will need to be validated in prospectively designed studies. Although smoking is a well‐known risk factor for lung cancer and has also been reported as a risk factor for VTE,^28^, ^29^ it did not increase the risk of PE in our lung cancer patients. In fact, it turned out to be a protective factor for lung cancer with PE, consistent with the pan‐cancer study by Au et al (9.2% vs. 21.1%, *p* < 0.01).^30^ After performing univariate analysis, we found that lung cancer patients who had positive CYFRA 21‐1, elevated WBC, AST, and higher ECOG PS score were predisposed to an increased risk of PE. However, after multivariate adjustment, we found that only a high PS score (PS > 1) was an independent risk factor for lung cancer patients with PE.

Nevertheless, there were a few shortcomings in our study that need to be addressed. Firstly, the study only included patients from the respiratory department, which resulted in the inclusion of mostly advanced TNM stage lung cancer patients. Secondly, the sample size was small, which might have impacted the generalizability of the findings. Additionally, due to the retrospective nature of the study, we faced difficulties in obtaining certain test results such as partial pressure of oxygen and NT‐proBNP, which hindered our ability to provide a comprehensive summary of the features.

## 5 CONCLUSION

The prevalence of PE in advanced lung cancer patients in this study was 1.08% and it was associated with a shortened OS. Dyspnea was the most dominant complaint, and poor PS score was found to be an independent risk factor for lung cancer with PE. Larger and prospective studies are needed to confirm our findings.

## AUTHOR CONTRIBUTIONS

**Yongkang Huang:** Conceptualization; writing (original draft); writing (review and editing). **Beilei Zhang:** Data curation; formal analysis; software; writing (review and editing). **Shiyuan Gao:** Data curation; formal analysis; software; writing (review and editing). **Ting Li:** Data curation; formal analysis; software; visualization; writing (review and editing). **Juan Du:** Data curation; writing (review and editing). Yajuan Qian: Validation; writing (review and editing). **Yufei Xing:** Validation; writing (review and editing). **Tong Zhou:** Writing (review and editing). **Minhua Shi:** Writing (review and editing). **Jian‐an Huang:** Conceptualization; funding acquisition; project administration; supervision. **Yixin Lian:** Conceptualization; funding acquisition; project administration; supervision.

## CONFLICT OF INTEREST STATEMENT

The authors have no conflicts of interest to declare.

## ETHICS STATEMENT

The study was carried out in accordance with the Code of Ethics of the World Medical Association (Declaration of Helsinki). The study protocol was approved by the ethics committee of the Second Affiliated Hospital of Soochow University (No. JD‐HG‐2022‐63). Informed consents were obtained from all patients or their families. The data underwent anonymization prior to statistical analyses and were handled in accordance with standard data protection regulations.

## Data Availability

Data about the clinical patients could be obtained from corresponding authors on a reasonable request.
